# Realizing a facile and environmental-friendly fabrication of high-performance multi-crystalline silicon solar cells by employing ZnO nanostructures and an Al_2_O_3_ passivation layer

**DOI:** 10.1038/srep38486

**Published:** 2016-12-07

**Authors:** Hong-Yan Chen, Hong-Liang Lu, Long Sun, Qing-Hua Ren, Hao Zhang, Xin-Ming Ji, Wen-Jun Liu, Shi-Jin Ding, Xiao-Feng Yang, David Wei Zhang

**Affiliations:** 1 State Key Laboratory of ASIC and System, Institute of Advanced Nanodevices, School of Microelectronics, Fudan University, Shanghai 200433, China; 2 State Key Lab of Silicon Materials, Zhejiang University, Hangzhou 310027, China; 3 Department of Optical Science and Engineering, Fudan University, Shanghai 200433, China

## Abstract

Nowadays, the multi-crystalline silicon (*mc*-Si) solar cells dominate the photovoltaic industry. However, the current acid etching method on *mc*-Si surface used by firms can hardly suppress the average reflectance value below 25% in the visible light spectrum. Meanwhile, the nitric acid and the hydrofluoric contained in the etching solution is both environmental unfriendly and highly toxic to human. Here, a *mc*-Si solar cell based on ZnO nanostructures and an Al_2_O_3_ spacer layer is demonstrated. The eco-friendly fabrication is realized by low temperature atomic layer deposition of Al_2_O_3_ layer as well as ZnO seed layer. Moreover, the ZnO nanostructures are prepared by nontoxic and low cost hydro-thermal growth process. Results show that the best passivation quality of the *n*^+^ -type *mc*-Si surface can be achieved by balancing the Si dangling bond saturation level and the negative charge concentration in the Al_2_O_3_ film. Moreover, the average reflectance on cell surface can be suppressed to 8.2% in 400–900 nm range by controlling the thickness of ZnO seed layer. With these two combined refinements, a maximum solar cell efficiency of 15.8% is obtained eventually. This work offer a facile way to realize the environmental friendly fabrication of high performance *mc*-Si solar cells.

Since year 2002, the multi-crystalline silicon (*mc*-Si) solar cells have become the mainstream products in most photovoltaic (PV) industrial production lines^1^,^2^. Recently, *mc*-Si solar cells have dominated more than 70% of the PV industry^3^. However, the average conversion efficiency (CE) of the *mc*-Si solar cells is still ~2% lower than that of the single crystalline silicon (*sc*-Si) based solar cell^4^. One main obstacle is the poor light trapping ability of the *mc*-Si based solar cells in the photo-active layers^5^. For the further CE improvement of the *mc*-Si solar cells, the front texture and antireflection coating play an important role by increasing light coupling into the active region of the devices. It is known that a pyramid texture is not applicable to the *mc*-Si wafer, although it can be formed on *sc*-Si wafer based on anisotropic alkali etching to minimize the optical reflection losses. The current texturing way for reducing surface reflectance on the *mc*-Si wafer used in industry is generally utilizing isotropic texturing in acidic solution containing nitric acid (HNO_3_) and hydrofluoric acid (HF) to form randomly overlapping hemispherical pits. However, this kind of etching method can hardly suppress the average reflectance (R) value below 25% in visible light spectrum^5^. That is much higher than the ~12% R value of the alkaline etched *sc*-Si surface. In addition, the HNO_3_ and HF contained in the texturing solution for *mc*-Si is both not eco-friendly as well as highly toxic to human skin and bone.

To efficiently suppress the optical reflection losses, the Si nanostructures (NS) formed by metal-catalyzed-etching (MCE)^6^,^7^,^8^, laser ablation^9^,^10^ and reactive-ion-etching (RIE)^5^,^11^,^12^,^13^,^14^ on *mc*-Si substrates have been proposed, which can achieve <5% reflectance over a wide spectral range (400–1100 nm) in combination with an SiN_x_ anti-reflection coating. Based on these methods, the obtained *mc*-Si solar cells with highest conversion efficiency (CE) of 18%^3^, 19.1%^10^ and 20.3%^12^ have been reported. Unfortunately, the MCE technique is [001] oriented silicon preferred^15^,^16^,^17^,^18^, which is not very effective on the *mc*-Si surface. Because the formed Si NS is unable to distribute homogeneously on the surface owning to the random crystalline characteristic of the *mc*-Si, the light trapping ability will be degrade^19^. Meanwhile, the AgNO_3_ contained in the etching solution is not cost effective. On the other hand, although the laser ablation and RIE method can be performed on both *sc*-Si and *mc*-Si surface, the high cost and low production rate makes them hard to be suitable for industrial production. More seriously, the required etching gas of SF_6_/Cl_2_^5^ is a threat to cause ozone hole or highly toxic to human. In recent years, the ZnO NS has aroused increasing attention for anti-reflection applications due to its low cost, good transparency, appropriate refractive index^20^,^21^,^22^,^23^,^24^. In contrast to Si NS, Green *et al*. reported that the ZnO NS can be grown on arbitrary substrates, regardless of the substrate crystallinit^25^. Lee *et al*. systematically investigated the light trapping ability of the ZnO NS with different morphologies^26^. Their results show that the optimized ZnO NSs as the anti-reflection layer for Si solar cell exhibit a superior light harvesting ability to that of the conventional SiN_x_ coating. More importantly, the ZnO NS can be grown in large scale by a simple and low cost hydro-thermal method^27^. In addition, the utilized reagents including zinc nitrate and hexamethylene tetraamine (HMT) to grow ZnO NS are nontoxic and at a very low concentration of 1–100 mM. The merits of the fabricating ZnO NS mentioned above pave a way for its environmental-friendly application on the *mc*-Si solar cells.

In the past decade, there have been application of ZnO NS on amorphous-Si (*a*-Si)^28^,^29^,^30^, *mc*-Si^31^,^32^, *sc*-Si^33^,^34^,^35^, GaAs^36^ and heterojunction^37^ solar cells, with CE ranges from 5.2% to 16.4%. In 2010, Chen *et al*. firstly utilized the vertically aligned ZnO nano-rod arrays as the anti-reflection layer in a *mc*-Si solar cell application^31^. Although an improved CE from 10.4% to 12.8% was obtained. It was still much lower than the ~18% CE value of the commercial available *mc*-Si solar cells. The main problem is the relatively low *V*_*oc*_ of ~550 mV Chen’s cell compared to those of SiN_x_ coated commercial cells (typically ~620 mV). This was mainly due to the high surface recombination rate on Si surface when ZnO NS grow directly on the Si wafer. To improve the Si surface passivation quality, Liu *et al*. prepared a thermal oxidation layer on the micro-pyramid *sc*-Si wafers before the formation of the ZnO NS^34^. This novel hierarchical structure shows a broadband reflection suppression in the 300–1200 nm rage, with an average weighted reflectance of 3.2%. An enhanced CE of 16% was achieved for the screen-printed *sc*-Si based solar cell. But the required SiO_2_ passivation layer formation temperature of 900 °C would led to huge energy consumption. Lin *et al*. reported an efficient broadband and omnidirectional light-harvesting scheme employing a ZnO nanorod/Si_3_N_4_-coated Si microgroove on 5-inch *sc*-Si solar cells. The processing temperature for the growth of the passivation layer was then decreased to ~450 °C through replacing the thermal grown SiO_2_ layer by plasma-enhanced-chemical-deposition (PECVD) grown SiN_x_ layer^35^. Unfortunately, the Si surface passivation quality was sacrificed, leading to a reduced solar cell conversion efficiency of 14.04%. It should be also noted that the preparations of the passivation layer and the ZnO seed layer were accomplished by the different machines for the above two mentioned solar cells. This would apparently increase the process complexity at the mass production level.

Encouragingly, the atomic-layer-deposition (ALD) technique has recently received great attention in the PV industry, which can be a potential solution to the problems. Taking the advantage of its relatively low growth temperate (around 200 °C)^38^,^39^, the heat budget to prepare the passivation layer by ALD can be reduced accordingly. It is reported that the ALD grown Al_2_O_3_ film exhibits the excellent passivation quality on the surface of the p-type Si solar cell owing to the fixed charge inside the prepared film^40^,^41^,^42^,^43^,^44^,^45^,^46^. Furthermore, the investigations reveal that the Al_2_O_3_ film also show good passivation ability on the n^+^ emitter of the p-Si solar cells^47^,^48^. But the ALD processing parameters including the Al_2_O_3_ film thickness as well as the followed annealing temperature have not been optimized. On the other hand, the seed layer of the ZnO NS can also be grown by ALD in the same reactor, which can simplify the fabrication process significantly. The hierarchical structure based on ZnO NS/Al_2_O_3_ passivation layer is then proposed to enhance the light trapping ability and improve the CE of the *mc*-Si solar cells.

In this work, a *mc*-Si solar cell based on ZnO NS and an Al_2_O_3_ passivation layer is demonstrated. As illustrated in Fig. 1, the ZnO NS are utilized to promote the light absorption into the *mc*-Si substrate. Meanwhile, the poor passivation ability of ZnO NS on the silicon surface is compensated by adding an Al_2_O_3_ passivation layer between the ZnO NS and the *mc*-Si. To realize the eco-friendly fabrication, the Al_2_O_3_ passivation layer as well as the seed layer for the growth of ZnO NS are all prepared by the low temperature ALD growth technique. After that, the ZnO NS are prepared by a conventional hydro-thermal growth method utilizing the nontoxic reagents. Both the Al_2_O_3_ layer passivation mechanism and the spectra absorption character of the ZnO NS are investigated deeply to achieve an optimized processing condition for the subsequent application in the *mc*-Si solar cell. It is found that there is a tradeoff between the Si surface dangling bond saturation level and the negative charge concentration in the Al_2_O_3_ passivation layer on the n^+^ -type *mc*-Si surface. The obtained solar cell with an optimized 12 nm-thick Al_2_O_3_ layer show an increment of 4.9% in the open circuit voltage (*V*_*oc*_) than that of the one with only ZnO NS. Furthermore, it is observed that the surface morphology as well as the antireflection character of the ZnO NS can be controlled by altering the thickness of the ZnO seed layer. A lowest average reflectance of 8.5% in 400~900 nm range can be achieved, leading to an increased solar cell performance in *J*_*sc*_ of 4.2 mA/cm^2^. Based on this hierarchical structure, a maximum CE value of 15.8% is obtained for the optimized *mc*-Si solar cell, which has a comparable performance to the previously reported *sc*-Si cell based on ZnO NS with a highest CE of 16.0%^34^. As a result, the findings of this work pave a facile way for realizing the eco-friendly fabrication and potential application of the high performance silicon solar cells in the future.

## Results and Discussion

### Optimization of the Al_2_O_3_ Passivation Layer

**Figure 2:**
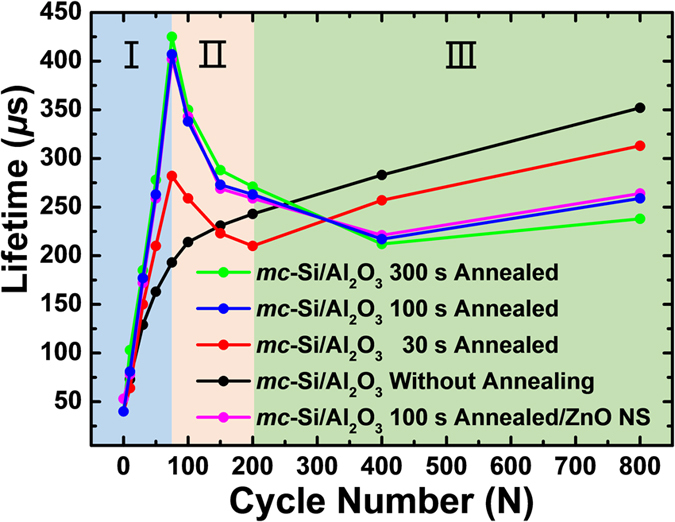
The minority carrier lifetimes of *mc*-Si/Al_2_O_3_ and *mc*-Si/Al_2_O_3_/ZnO NS samples as a function of annealing time and Al_2_O_3_ thickness.

To realize the best passivation quality of the Al_2_O_3_ film, the minority carrier lifetime (τ) on the 70 Ω per square n^+^ doped *mc*-Si surface are investigated as a function of the Al_2_O_3_ film thickness (determined by the ALD growth cycles) as well as the annealing duration of the Al_2_O_3_/*mc*-Si stacks. The results are shown in Fig. 2. As is evidently shown in Fig. 2, the unannealed *mc*-Si/Al_2_O_3_ stacks and the annealed ones exhibit quite different variation tendency in τ. The τ of the unannealed stack exhibit a low τ of 40 μs. Then the τ value increases monotonically to 352 μs as the growth cycle of the Al_2_O_3_ reaches 800. While for the annealed stacks, the value of the Al_2_O_3_ growth cycle determines three different regions of the τ performance. For a given annealing duration, the τ increases (Region I) as the Al_2_O_3_ growth cycle increases from 0 to 75. A peak value of 425 μs is obtained by the 300 s annealed sample as the growth cycle reaches 75. Then the τ value decreases (Region II) as the growth cycle further increases from 75 to 200. Finally, a slight increase trend in τ is observed again at highest growth cycle (Region III). At each value of the growth cycle, the prolonged annealing time leads to a higher τ value in Region I and II. In contrast, the opposite trend is observed in Region III. For example, at the growth cycle of 75, the τ value of the samples increases from 193 μs to 425 μs as the annealing duration increases from 0 to 300 s. Oppositely, at the growth cycle of 800, the τ value decreases from 352 μs to 238 μs as the annealing duration increases. Furthermore, the passivation effect of the structure of Si/Al_2_O_3_/ZnO NS with 100 s Al_2_O_3_ annealing time is further checked by measuring its minority carrier lifetime. As shown in the violet line of Fig. 2, the minority carrier lifetime changes little after the formation of the ZnO NS on the *mc*-Si/Al_2_O_3_ sample. It indicated that the subsequent ZnO NS preparation has little effect on the passivation quality of the Al_2_O_3_ on the Si surface. As a result, the subsequent ellipsometry and C-V tests are just conduct on the substrates with only Al_2_O_3_ coating for obtaining more accurate SiO_2_ thickness and flat band voltages. Given consideration to both passivation quality as well as the heat budget, the 100 s annealed stack with 75 cycle Al_2_O_3_ growth (~12 nm thick and with a relatively high τ of 407 μs) is selected as the optimum process conditions for the following solar cell fabrication.

**Figure 3:**
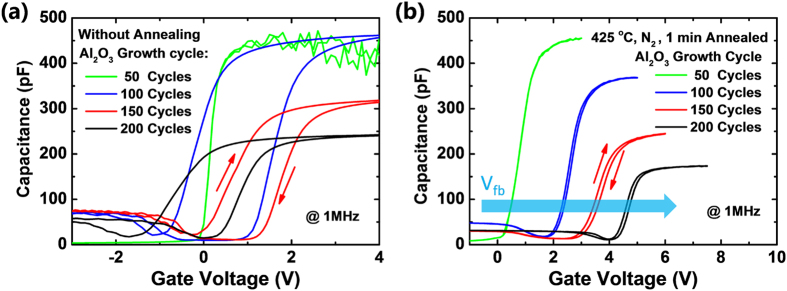
The *C-V* curves of the *mc*-Si/Al_2_O_3_ capacitors in which the Al_2_O_3_ layer growth cycle increases from 50 to 200, (**a**) without annealing (**b**) 425 °C annealed in N_2_ environment with a duration from 0 to 300 s.

Further investigations are made to understand the passivation mechanism of the Al_2_O_3_ film with different thickness on the n^+^ doped *mc*-Si surface. During the annealing treatment on Si/Al_2_O_3_ stack, the excess oxygen inside the Al_2_O_3_ layer will diffuse to the Al_2_O_3_/Si interface and forms a SiO_2_ interlayer^49^. The SiO_2_ layer brings chemical passivation effect by saturating the dangling Si bonds on *mc*-Si surface^41^,^45^,^49^. As a result, the thickness of the SiO_2_ interfacial layer is analyzed. Before the annealing treatment, owning to the low growth temperature (200 °C) of the ALD-Al_2_O_3_, the SiO_2_ layer is unable to be recognized by the ellipsometer. Table 1 shows the thickness evolution of the SiO_2_ interfacial layer of the annealed Al_2_O_3_ samples with growth cycles increased from 50 to 200. After the annealing procedure, the thickness of the SiO_2_ layer evidently increases. As the Al_2_O_3_ growth cycles increases from 50 to 100, the thickness of the SiO_2_ increased by 57% (1.84 to 2.81 nm). At this moment, the increment of SiO_2_ (8.84 to 12.98 nm) coordinates with that of the Al_2_O_3_ film. However, as the growth cycles increased from 150 to 200 cycles, the thickness increment of the SiO_2_ greatly reduced to 7%, and 1%, respectively. It can be predicted that the thickness of the SiO_2_ layer will stand-still at ~3.1 nm at higher growth cycles. This phenomenon can be explained by the annealing condition of the all the Al_2_O_3_ samples are the same. So, the amount of the oxygen which can diffuse to the Si/Al_2_O_3_ interface is limited. Thus, the chemical passivation effect due to the formation of SiO_2_ layer is most effective at a relative low Al_2_O_3_ growth cycle ranging from 0 to 100. After that, the negative charge concentration of the Al_2_O_3_ films are investigated. Because the negative charges can lead to field passivation effect, which can impact the electron density at the *mc*-Si surface^40^,^46^,^50^. Figure 3a,b describes the C-V characteristics of the Mo/Al_2_O_3_/n^+^ -type *mc*-Si capacitor, in which the Al_2_O_3_ growth cycles ranges from 50 to 200. As shown in Fig. 3a, before annealing, the 50 cycles grown Al_2_O_3_ film exhibit electric break-down in accumulation capacitance owning to the relatively low film thickness. The C-V curves of the samples with 100–200 growth cycles exhibit a wide flat band hysteresis window, indicating a high interface trap density. We speculate this phenomenon is mainly caused by the existence of the Si dangling bonds at the *mc*-Si/Al_2_O_3_ interface. This observation corresponds with the poor τ performance of the unannealed Al_2_O_3_ samples shown in Fig. 2. After the annealing treatment, the forged SiO_2_ interfacial layer saturated most of the dangling Si bonds. In sequence, the flat band hysteresis windows of the samples are greatly narrowed in Fig. 3b. More importantly, as the Al_2_O_3_ growth cycles increases, their corresponding flat band voltage shifts toward positive direction, indicating a gradually increased negative charge concentration. One part of the negative charges come from the formation of the tetrahedrally coordinated Al site in the Al_2_O_3_ film after the annealing. Another part of the negative charges comes from the SiO_2_ interfacial layer^45^. For the p^+^ type silicon passivation, electrons are the minority carriers. The negative charges inside the Al_2_O_3_ film plays a positive role as it can prevent the electrons from diffusing to the surface of the p^+^ type Si surface. In our case, the negative charges plays a negative role, because it will accelerate the diffusion of the holes to the n^+^ type Si surface. The τ evolution in the three regions of Fig. 2 can be explained by the combined affection of the two passivation effects mentioned above. In region I, the chemical passivation by the formation of SiO_2_ layer dominates the passivation mechanism. Because at this moment, the thickness of the SiO_2_ increases quickly. Thus, more and more Si dangling bonds are saturated. Meanwhile, the negative charge concentration inside the passivation layer is low, the corresponding negative influence is minimized. As a result, the τ value increases quickly in region I. While in region II, the growth of the SiO_2_ layer stops, but the negative charge inside the Al_2_O_3_ layer continue to increase. In this situation, the field passivation effect plays the main role. Unfortunately, as is mentioned above, the negative charge concentration will accelerated the recombination rate on the n^+^ Si surface, the τ value decreases. At even higher Al_2_O_3_ growth cycles in region III, the increment of negative charge concentration is mainly contributed by the formation of Al_2_O_3_ at outer side film, which can hardly influence the holes inside the n^+^ type *mc*-Si. In consequence, the τ value gradually increases as a result of the increased thickness of the passivation layer. Results show that a thin Al_2_O_3_ layer can saturate most of the Si dangling bonds while eliminating the negative influence of negative charges on n^+^ doped Si. As a result, the best passivation quality is obtained.

### Optical Optimization of the ZnO NS

**Figure 4:**
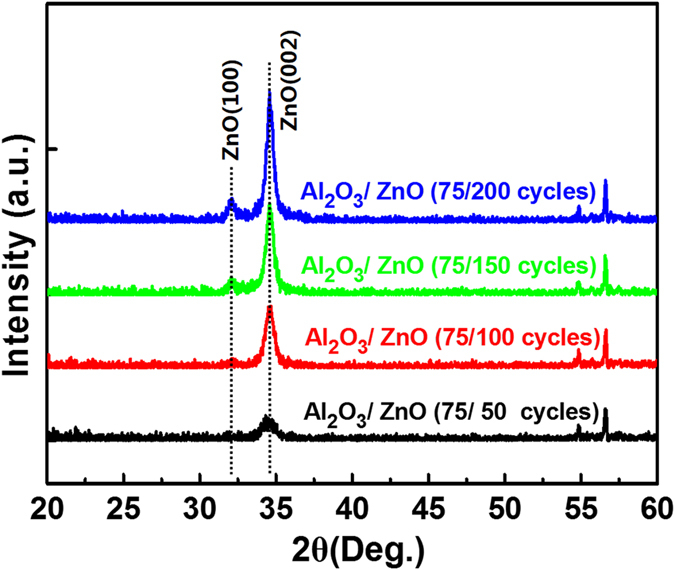
The XRD pattern of the ZnO seed layers with growth cycles increase from 50 to 200, respectively.

**Figure 5:**
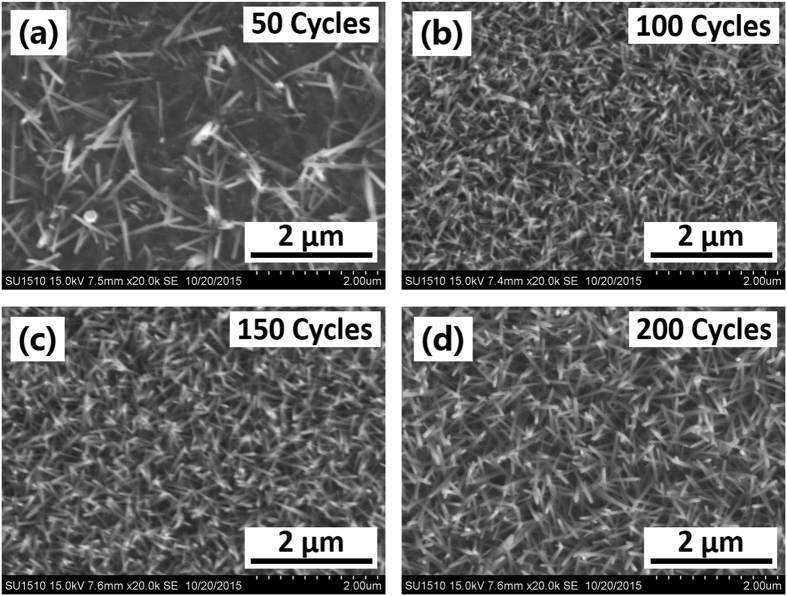
(**a–d**) Morphology evolution of the ZnO NS synthesized from the ZnO seed layers indicated in Fig. 4.

There have been many investments on anti-reflection character of the ZnO NS synthesized from spin-coated seed layer^25^,^26^,^27^. However, the film thickness cannot be controlled precisely by spin-coating. To overcome this difficulty, the precise film thickness control of the ZnO seed layer is achieved by ALD growth in this work, taking the advantage of the layer-by-layer growth mechanism of the ALD. The anti-reflection character of the ZnO NS synthesized from ALD grown seed layer with different thickness is systematically investigated in this work. At first, the crystallization behavior of the ALD grown ZnO seed layer is investigated because it will greatly affect the surface morphology of the later grown ZnO NS^25^. Figure 4 shows the XRD patterns of the ALD-ZnO seed layers with different growth cycles (ranging from 50 to 200) prepared on the surface of the optimized *mc*-Si/Al_2_O_3_ stack. The seed layer based on 50 growth cycles exhibit a weak ZnO (002) peak at 2θ = 34.4°, which shows that the as-deposited seed layer is mainly amorphous. As the seed layer growth cycle reaches 100, the ZnO (002) peak become stronger, indicating some small ZnO crystal grains started to form inside the seed layer. The calculated ZnO crystal grains size inside the seed layer using the Scherrer formula^51^ are summarized in Table S1 in the supporting information. The full-width half-maximum (FWHM) of the ZnO (100) peak decreases as the growth cycle increases, demonstrating a gradually increased grain size of the ZnO crystal form 14.2 to 31.7 nm in the seed layer. Figure 5a–d show the morphology evolution of the ZnO NS synthesized from the seed layers demonstrated in Fig. 4. As shown in Fig. 5a, the ZnO NS prepared on the 50 cycles grown seed layer exhibit a disordered morphology and distribution. One reason is the poor crystalline degree of the seed layer as indicated in Fig. 4. Another reason is that the seed layer is relatively thin, which is unable to efficiently avoid the inhibiting effect of the Al^3+^ (contained beneath the Al_2_O_3_ layer) on the growth of the ZnO NS^52^. The ZnO NS started to exhibit an ordered morphology after the seed layer growth cycle reaches 100. The TEM image of one ZnO NS shown in Fig. S1 in the supporting information exhibit that the as-grown ZnO NS are single crystalline. As the seed layer growth cycle increases from 100 to 200, the obtained ZnO NS exhibit an increase in diameter and length. On the contrary, the density of the ZnO NS is reduced. The increased diameter is owning to the increased grain size of the ZnO crystal in the seed layer. Because the single crystal epitaxial growth of the ZnO NS are most likely to start from the crystal grains inside the seed layer. We speculate that the increased ZnO crystal grain size is achieved by linking the nearby small grains together. Thus, the number of the grains reduces, leading to the reduced density of the ZnO NS. As fewer NS can be grown within a certain area, the growth of each wire-shaped NS is accelerated, leading to the increased length of the NS. Results show that the surface morphology of the ZnO NS can be controlled by altering the thickness of the ALD grown ZnO seed layer.

**Figure 6:**
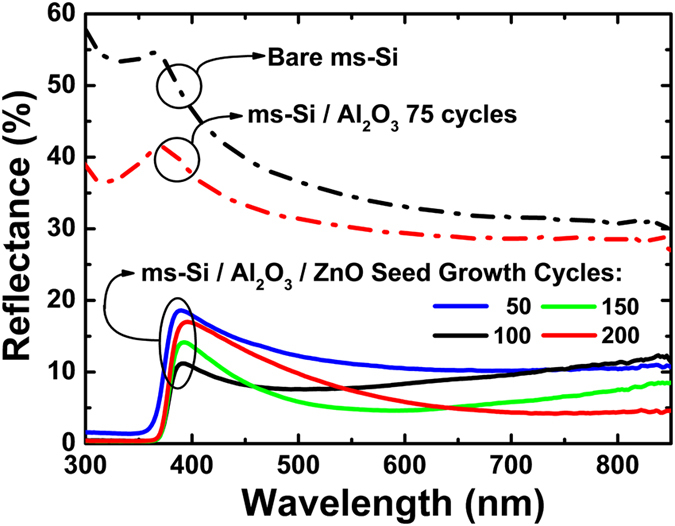
Reflectance spectra of the bare *mc*-Si, the optimized *mc*-Si/Al_2_O_3_ stack, the *mc*-Si/Al_2_O_3_/ZnO NS stacks based on ZnO seed layer growth cycle ranging from 50 to 200.

To evaluate the light trapping ability, the reflectance spectra of the bare *mc*-Si, the optimized *mc*-Si/Al_2_O_3_ stack, the *mc*-Si/Al_2_O_3_/ZnO NS stack prepared in Fig. 5a–d are shown in Fig. 6. Significantly, more than 35% of the incident light is reflected away from the bare *mc*-Si surface. Deposition of the Al_2_O_3_ layer only helps a little, the reflectance loss still excess 30%. It is encouraged that the reflectance is largely suppressed after the growth of the ZnO NS. It is not surprising that the ZnO NS synthesized from the 50 cycles grown seed layer show the highest reflection, because the distribution of the ZnO NS is inhomogeneous (indicated in Fig. 5a). As the seed layer growth cycle increases from 100 to 200, the lowest reflectance value of each *mc*-Si/Al_2_O_3_/ZnO NS stack gradually decreases (from 7.6% of 100 cycles seed layer growth to 4.3% of 200 cycles seed layer growth). Meanwhile, the lowest reflectance value point shifts from the short wavelength toward the long wavelength (from 7.6% of 100 cycles seed layer growth to 4.3% of 200 cycles seed layer growth). The reduced lowest reflectance value is owing to the increased length of the NS, which enhances the multiple reflections of the incident light. We speculate that the spectrum shift of the lowest reflectance value point is as a result of the increased thickness of the ZnO seed layer. For single anti-reflection layer, the relationship between the film thickness (d) and the lowest reflectance wavelength can be explained as:


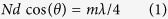


where *N* is the refractive index of the film, *d* is the film thickness, *θ* is the angle of the incident light, *m* is any natural number. From this equation, it is evident that the increased film thickness will lead to the “red shift” of *λ*. This trend corresponds with our observations. The calculated solar energy weighted (AM 1.5) reflectance (R_w_) of the nanostructured surface with seed layer growth cycle ranges from 100 to 200 are 9.3%, 8.5% and 8.7% respectively. Results show that the seed layer growth cycle of 150 is the optimum value for the fabrication of the solar cells.

**Figure 7:**
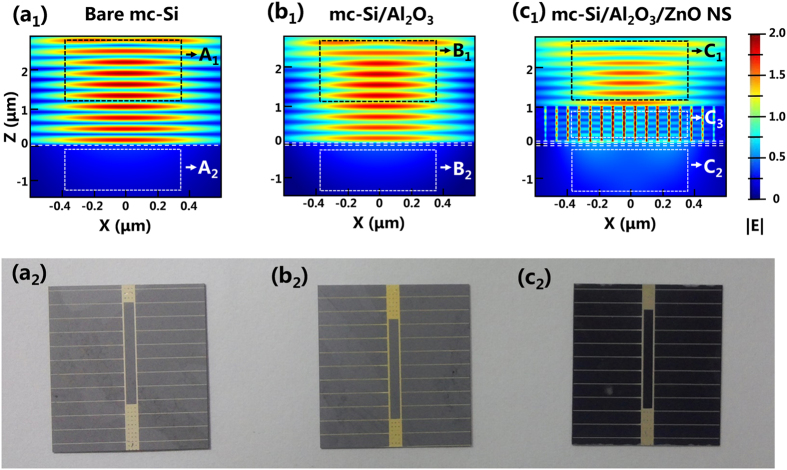
Simulated cross-sectional light intensity |E_y_| distribution at 550 nm wavelength on (**a**_**1**_) bare *mc*-Si surface, (**b**_**1**_) optimized *mc*-Si/Al_2_O_3_ stack, (**c**_**1**_) optimized *mc*-Si/Al_2_O_3_/ZnO NS stack. (**a**_**2**_**–c**_**2**_) photographs of the fabricated *mc*-Si solar cells based on the bare *mc*-Si surface, the optimized *mc*-Si/Al_2_O_3_ stack, the optimized *mc*-Si/Al_2_O_3_/ZnO NS stack, respectively.

Two dimensional (2D) finite difference time domain (FDTD) analysis is carried out to gain insight into the light harvesting mechanism of the optimized *mc*-Si/Al_2_O_3_/ZnO NS stack. The bare *mc*-Si surface as well as the optimized *mc*-Si/Al_2_O_3_ stack are also added for comparison. In the simulation, the wavelength of the incident light is chosen to be 550 nm, which is close to the peak irradiance in solar spectrum. The dimension of the Al_2_O_3_ layer (12 nm thick, n = 1.65 at 550 nm), the ZnO seed layer (30.5 nm, n = 2.05 at 550 nm) are obtained from ellipsometry measurement. The dimension of the ZnO NS (1 μm in length, 60 nm in diameter, 70 nm in period) are averaged from the SEM image show in Fig. 5c. The light intensity distribution (|E_y_|) for the three structures mentioned above are shown in Fig. 7a1–c1. The strong |E_y_| on both of the *mc*-Si (Region A_1_) and the *mc*-Si/Al_2_O_3_ stack (Region B_1_) indicates a high surface reflectance, confirming with the reflectance results shown in Fig. 6. It is evidently shown in Region C_1_ that the the intensity of reflected light is greatly reduced after the formation of the ZnO NS. The strong |E_y_| distribution at the Region C_3_ indicating the ZnO NS play a crucial role in suppressing light reflection. One reason is that ZnO has a relatively appropriate refractive index of ~2 in visible light spectrum^53^. Meanwhile, the NS morphology provides a density-graded interference between the air and the substrate^26^,^54^. Moreover, the effective path length of the incident light is prolonged by multiple reflection effect between the NS. These three effects further boost the light trapping ability of the ZnO NS. As shown in Region C_3_, the bright field inside each ZnO NS reveals that most of the incident light couples into the cylinder-shaped NS. The red field shown in region C_3_ suggest that another part of the incident light as well as the escaped light from the NS undergo multiple bounces between the nearby NS. At each bounce, more light is coupled into the ZnO NS. Owing to the wide bandgap character of both ZnO and Al_2_O_3_, these two materials are highly transparent in visible light spectrum. Thus, the light couples into the ZnO NS are then transmitted to the ZnO seed layer and the beneath Al_2_O_3_ layer, eventually absorbed by the *mc*-Si. Benefited from the excellent anti-reflection ability of the ZnO NS, the |E_y_| distribution in region C_2_ is obviously stronger than that in Region A_2_ and B_2_, demonstrating an enhanced light absorption of the *mc*-Si substrate. Figure 7a2–c2 show the vision image of the *mc*-Si solar cells based on the three surfaces mentioned above. Comparing with the cells in Fig. 7a2,b2, the cell in Fig. 7c2 is black in color, reconfirming the wide-band photon capturing ability of the ZnO NS in visible light spectrum.

### Photovoltaic Performance

**Figure 8:**
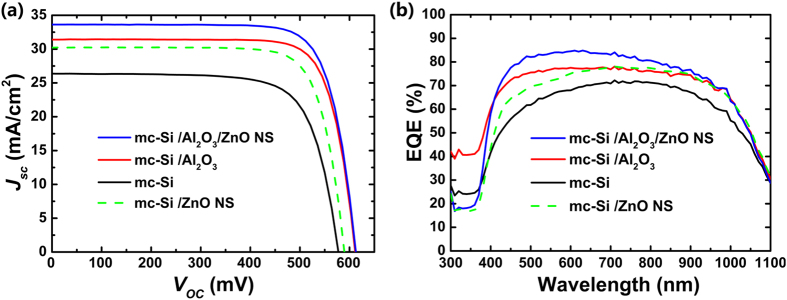
Photovoltaic performance of the *mc*-Si cells with different surface structures: (**a**) J-V, (**b**) EQE.

To verify the effectiveness of the optimized *mc*-Si/Al_2_O_3_/ZnO NS stack for photovoltaic applications, the corresponding solar cells are subsequently fabricated. The detected J-V curves are shown in Fig. 8a, the obtained cell parameters are summarized in Table 2. The bare *mc*-Si solar cell exhibit a poor *V*_*oc*_ and *J*_*sc*_ performance, which is related to the high surface recombination rate and consistent with its low τ of ~43 μs shown in Fig. 2. The cell based on the optimized *mc*-Si/Al_2_O_3_ stack exhibit a simultaneous increase in *V*_*oc*_ and *J*_*sc*_, leading to an evidently improved CE. Our previous work indicate that the improvement is as a result of suppressed surface recombination rate on *mc*-Si by Al_2_O_3_ passivation (echo with the τ increment in Region I of Fig. 2). A further increment in *J*_*sc*_ of ~6.1% is observed by the cell based on the optimized *mc*-Si/Al_2_O_3_/ZnO NS stack. The improvement in *J*_*sc*_ is mainly contributed by the significantly lowered surface reflectance on *mc*-Si/Al_2_O_3_ stack after the formation ZnO NS (indicated in Fig. 6), leading to an increase in photon generated carriers. A maximum conversion efficiency of 15.8% is achieved by the optimized *mc*-Si solar cell with two functional layers. Meanwhile, the *V*_*oc*_ performance of the solar cell with *mc*-Si/Al_2_O_3_/ZnO NS and *mc*-Si/Al_2_O_3_/ZnO NS stack are identical. It indicated that the passivation quality of the Al_2_O_3_ on Si surface has not degraded very much after the ZnO NS growth, which agrees with the minority carrier lifetimes shown in Fig. 2. Moreover, the cell based on *mc*-Si/ZnO NS is also fabricated for comparison. Although an improved *J*_*sc*_ of the cell is also observed, the CE is limited by the low *V*_*oc*_ value. It can be concluded that the Al_2_O_3_ layer and the ZnO NS can compensate for each other in surface passivation and light trapping. Both of them are indispensable for achieving the maximum CE of the *mc*-Si solar cell in this work.

The external quantum efficiency (EQE) analyzation is performed on the representative cells listed in Table 2. The obtained results are shown in Fig. 8b. Comparing with the solar cell based on bare *mc*-Si, the Al_2_O_3_ passivated cell exhibit an enhanced photon conversion ability in the whole 300–1100 range owning to the suppressed surface recombination rate. After the ZnO NS are further added on the Al_2_O_3_ film, the obtained cell exhibit a reduced photon conversion rate in the range of 300–400 nm. This can be explained by the band edge absorption of the ZnO for short wavelength photons. Therefore, less photons in that range can be absorbed by the *mc*-Si substrate. It is encouraged that the EQE of the cell based on the Al_2_O_3_/ZnO NS stack show an evident increase in 450–700 nm range. The increase is owing to the improved anti-reflection ability in the visible spectrum by the application of the ZnO NS. Fortunately, the solar irradiation mainly concentrates in that range^55^, leading to a net increase in *J*_*sc*_ performance of 1.9 mA/cm^2^ of the obtained cell. Comparing with the cell with the bare *mc*-Si surface, the cell based on the *mc*-Si/ZnO NS also exhibit an improved EQE performance. However, the improvement is limited by the high surface recombination rate owing to the poor passivation quality of the ZnO. Therefore, the cell based on the *mc*-Si/ZnO NS show lower EQE in high solar energy range (450–700 nm) than the Al_2_O_3_ passivated cell, leading to the overall poor *J*_*sc*_ performance shown in Table 2. Results show that if the Al_2_O_3_ layer is removed away from the *mc*-Si/Al_2_O_3_/ZnO NS stack, the merit in photon capturing of the ZnO NS will be overwhelmed by the poor passivation quality of the ZnO seed layer on the *mc*-Si surface.

To further prove the effectiveness of the *mc*-Si solar cell based on the Al_2_O_3_/ZnO NS stack demonstrated in this work, comparisons are made among the a-Si, *sc*-Si and *mc*-Si solar cells based on the ZnO NS reported in recent years. The photovoltaic performance of these cells are listed in Table 3. Although the a-Si solar cells developed by Nowak *et al*.^28^,^29^ exhibit relatively low CEs below 10%, the fabrication procedure was simple and cost-effective. For the *mc*-Si solar cells, the *CE* of the *mc*-Si solar cell with ZnO NS anti-reflection surface demonstrated by Chen *et al*.^31^ was limited by its relatively low *V*_*oc*_ of ~500 mV (typically >610 mV for commercial *sc*-Si cells). The identical results were also obtained by Aurang *et al*. in *sc*-Si solar cell applications^33^. This was owing to the poor passivation quality of the ZnO on Si surface as mentioned above. In the current work, this problem was settled by adding an Al_2_O_3_ interfacial layer between the *mc*-Si and the ZnO NS as described in Table 2, which is similar to those demonstrated in refs 34 and 35. The ALD-Al_2_O_3_ passivation layer have shown several merits over the thermal-SiO_2_^34^ and the PECVD-SiN_x_^35^ films. As compared to the thermal-SiO_2_, the deposition temperature of the passivation layer can be reduced greatly from 850 °C to 200 °C by utilizing the ALD-Al_2_O_3_. Moreover, the Al_2_O_3_/ZnO seed layer stacks can be prepared in an ALD chamber. On the other hand, the ALD-Al_2_O_3_ layer was pin-hole free. Comparing with the PECVD-SiN_x_ thin film. During the hydro-thermal growth procedure of the ZnO NS, the pin-hole free passivation layer can prevent the reaction solution from reaching the Si/front electrode area more efficiently. This is beneficial for obtaining high quality solar cells with good *V*_*oc*_ and *FF* performance. However, The *J*_*sc*_ of the cell demonstrated in this work was not as high as that of the others. Because there is no surface texturing process on *mc*-Si in this work. On the positive side, the usage of highly toxic or corrosive chemicals such as HNO_3_ and HF was avoided, which is very important for realizing the eco-friendly production. Fortunately, the *J*_*sc*_ loss of the cells in this work was largely compensate by a relatively high *FF* value, which is as a result of improved ohmic contact formation quality of the front electrodes. This is realized by changing the front electrode screening and firing steps prior to the Al_2_O_3_ passivation. Eventually, a maximized conversion efficiency of 15.8% is achieved by the multi-crystalline solar cell with the optimized Al_2_O_3_/ZnO nanostructures stack, which exhibit comparable performance to the previously reported *sc*-Si cell based on ZnO nanostructures with highest conversion efficiency of 16.0%^34^. It is noted that the Al_2_O_3_/ZnO NS stack demonstrated in this work offers a potential way for realizing the eco-friendly fabrication of high performance *mc*-Si solar cells.

## Conclusion

In summary, a multi-crystalline solar cell with two functional layers is demonstrated by introducing ZnO nanostructures and an Al_2_O_3_ spacer layer. The eco-friendly fabrication is achieved by the low temperature deposition of an Al_2_O_3_ and a ZnO seed layer utilizing the atomic-layer-deposition technic. The ZnO nanostructures are prepared by and nontoxic hydro-thermal growth. For passivating the n^+^ -type multi-crystalline Si, most of the dangling bonds on the multi-crystalline Si surface can be saturated by a 12 nm thick Al_2_O_3_ film. Meanwhile, the adverse impact of the negative charge concentration inside the Al_2_O_3_ film is minimized. The imperfectness of the Al_2_O_3_ layer in anti-reflection is conquered by a further growth of ZnO nanostructures. Results show that the ZnO nanostructures synthesized from the seed layer with optimized thickness exhibit excellent photon harvesting ability in the visible light range, leading to an evident increase in short circuit current of the solar cells. Eventually, a maximized conversion efficiency of 15.8% is achieved by the multi-crystalline solar cell with the optimized Al_2_O_3_/ZnO nanostructures stack, which exhibit comparable performance to the previously reported single-crystalline Si cell based on ZnO nanostructures with highest conversion efficiency of 16.0%. Consequently, the proposed hierarchical nano structure in this work pave a facile way for realizing the eco-friendly fabrication and potential application of the high performance silicon solar cells in the future.

## Methods

### Preparation of the Al_2_O_3_/ZnO NS stack

The Al_2_O_3_ passivation layer and the ZnO seed layer were deposited by thermal ALD (Beneq TFS-200) at 200 °C. For the Al_2_O_3_ growth, the precursors were trimethylaluminium (TMA) and H_2_O. Then, the as-deposited Al_2_O_3_ films were annealed under a N_2_ environment at 425 °C in a rapid thermal annealing (RTA) furnace (Annealsys AS-ONE). After that, the ZnO seed layer was deposited, utilizing diethylzinc (DEZ) and H_2_O as the precursors. Finally, the ZnO NS were grown by hydro-thermal method at 80 °C. The utilized reactors were zinc nitrate and hexamethylene HMT, with equal concentration of 25 mM. The growth duration was 8 hours.

### Fabrication of the solar cell

Commercial grade p-type *mc*-Si wafers with a resistivity of 1–3 Ω∙cm were used as the starting substrate. At first, the wafers are immersed in 20 wt. %, 80 °C NaOH solution for 5 min for removing saw damages. Then, an n^+^ emitter with a sheet resistance of 70 Ω per square was formed using liquid POCl_3_ diffusion. The formed phosphor silicon glass was removed by immersing the wafers in diluted HF solution. After that, the front/back electrode was formed by screen- printing of silver/aluminum paste. The area-fraction on the surface was ~9% (the power loss induced by grid shadow was not excluded in the *J*_*sc*_ and *CE* calculation), the active area of each cell was 3.24 cm^2^. The front/rear electrode metallization were realized by annealing the samples in the RTA furnace in air with an peak temperature of ~720 °C. The samples were then shortly immersed into diluted HF to remove the oxide layer and contaminant formed on the front surface of the samples during the formation/annealing step of the electrodes. Then, the Al_2_O_3_/ZnO NS stack were prepared on the front surface of the cells.

### Sample characterization

The minority carrier lifetime on the *mc*-Si surface was obtained using a life time tester (Semilab WT-1000). The C-V curves were measured by a semiconductor device analyzer (Agilent B1500A). The XRD measurement was carried out in Bruker D8 system. The surface morphologies were characterized by a SEM (Hitachi SU1510). The reflectance spectra were measured using a spectrometer (Shimadzu UV3600) equipped with an integrating sphere. The solar cell performance was obtained under a standard 1-sun illumination with a sun simulator (Oriel-940401A) and a sourcemeter (Keithley-2400). The data obtained was based on an average of about 6 wafers/cells.

## Additional Information

**How to cite this article**: Chen, H.-Y. *et al*. Realizing a facile and environmental-friendly fabrication of high-performance multi-crystalline silicon solar cells by employing ZnO nanostructures and an Al_2_O_3_ passivation layer. *Sci. Rep.* **6**, 38486; doi: 10.1038/srep38486 (2016).

**Publisher’s note:** Springer Nature remains neutral with regard to jurisdictional claims in published maps and institutional affiliations.

## Supplementary Material

Supplementary Information

## Figures and Tables

**Table 1:** Thickness evolution of the SiO_2_ interface layer and the Al_2_O_3_ layer after the annealing treatment.

Al>_2_O_3_growth cycles	SiO_2_ thickness (nm)	Al_2_O_3_ thickness (nm)
50	1.84	8.84
100	2.81	12.98
150	3.01	19.21
200	3.07	24.25

**Table 2:** Solar cell efficiency and J-V parameters of the *mc*-Si cells with different surface structures.

>Sample	*V*_oc_ (mV)	*J*_sc_ (mA/cm^2^)	*FF*(%)	*CE*(%)
Bare *mc*-Si	579	26.4	73.9	11.3
*mc*-Si/Al_2_O_3_	611	31.4	77.4	14.8
*mc*-Si/Al_2_O_3_/ZnO NS	613	33.6	76.9	15.8
*mc*-Si/ZnO NS	590	30.2	77.6	13.8

**Table 3:** Literature comparison in photovoltaic performance of the Si solar cells based on ZnO NS published in recent years.

Year	Substrate	Surface texture	Passivation layer	*V*_oc_ (mV)	*J*_sc_ (mA/cm^2^)	*FF*(%)	*CE*(%)
2014	a-Si	/	/	831	10.4	60.2	5.2^28^
2014	a-Si	/	/	810	13.14	65.4	6.9^29^
2013	*sc*-Si	NaOH	/	580	28.6	74	12.7^33^
2012	*sc*-Si	NaOH	SiO_2_	616	35.3	73.4	16.0^34^
2012	*sc*-Si	NaOH	SiN_x_	609	38.5	69	14.0^35^
2010	*mc*-Si	HF/HNO_3_	/	~506	~36	70	12.8^31^
This work	*mc*-Si	/	Al_2_O_3_	613	33.6	76.9	15.8

**Figure 1:**
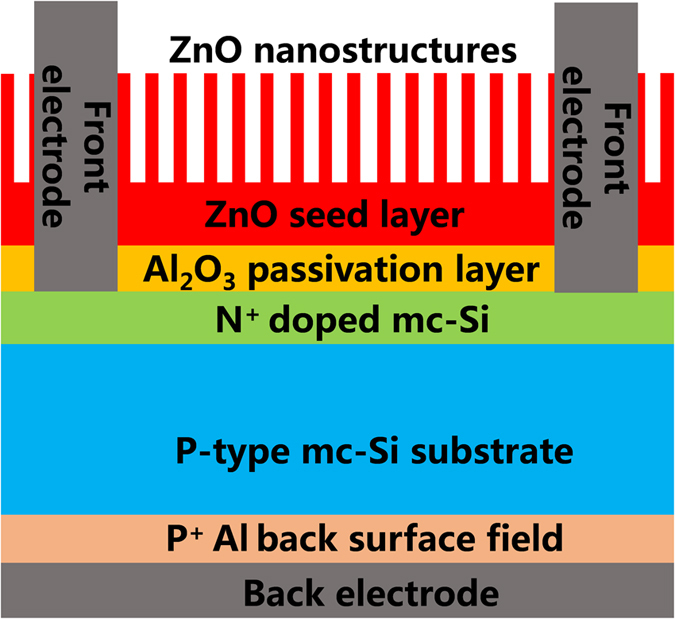
The schematic diagram of the *mc*-Solar cell based on the Al_2_O_3_ layer and ZnO NS demonstrated in this work.
