# Supplementary material for: Realizing a facile and environmental-friendly fabrication of high-performance multi-crystalline silicon solar cells by employing ZnO nanostructures and an Al2O3 passivation layer
Source: Sci Rep. 2016 Dec 7;6:38486. doi: 10.1038/srep38486 (PMC5141414; doi:10.1038/srep38486)
Supplement: Supplementary Information [file srep38486-s1.doc]

**Supporting Information**

**Realizing a facile and environmental-friendly fabrication of high-performance multi-crystalline silicon solar cells by employing ZnO nanostructures and an Al_2_O_3_ passivation layer**

Hong-Yan Chen^1,*^, Hong-Liang Lu^1,3,*^, Long Sun^1^, Qing-Hua Ren^1^, Hao Zhang^2^,

Xin-Ming Ji^1^, Wen-Jun Liu^1^, Shi-Jin Ding^1^, Xiao-Feng Yang^1^ & David Wei Zhang^1^

^1^State Key Laboratory of ASIC and System, Institute of Advanced Nanodevices,

School of Microelectronics, Fudan University, Shanghai 200433, China

^2^Department of Optical Science and Engineering, Fudan University, Shanghai

200433, China

^3^State Key Lab of Silicon Materials, Zhejiang University, Hangzhou 310027, China

*These authors contributed equally to this work.

Tel.:+86 021 65642457

Correspondence and requests for materials should be addressed to Prof. Hong-Liang Lu (honglianglu@fudan.edu.cn) or Prof. David Wei Zhang (dwzhang@fudan.edu.cn)


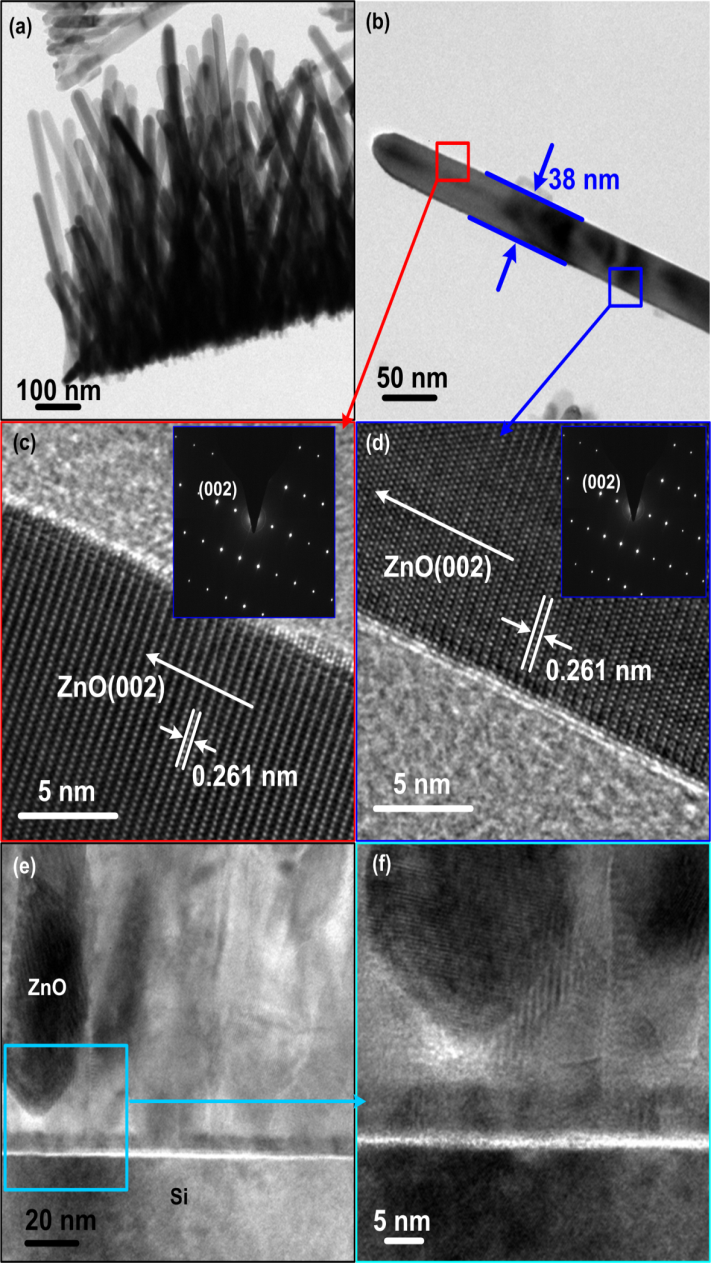


**Fig. S1** TEM image of the ZnO nanostructures synthesized from the 50 cycles grown seed layer, (a) and (b) low-magnification, (c) and (d) high-magnification.

The crystal grain size (D) of the ZnO seed layer was estimated by the Scherrer formula:

$$D=k\lambda/\beta\cos\theta$$

where *D* is the averaged crystallite size, *k* (=0.89) is a constant, *λ* (=1.54 Å in this case for Cu Kα radiation) is the X-ray wavelength, *β* is the full width at half maximum (FWHM) of the peaks, and *θ* is the Bragg angle. The FWHM values and the calculated average crystal grain size for ZnO (002) orientation were listed in table S1.

**Table S1.** Thickness evolution of the SiO_2_ interface layer and the Al_2_O_3_ layer after the annealing treatment.

| ZnO growth cycles | FWHM  (^o^) | Averaged crystal grain size  (nm) | |
| --- | --- | --- | --- |
| 50 | 0.58 | | 14.19 |
| 100 | 0.46 | | 17.89 |
| 150 | 0.35 | | 23.51 |
| 200 | 0.26 | | 31.65 |
